# Degree-Based Graph Entropy in Structure–Property Modeling

**DOI:** 10.3390/e25071092

**Published:** 2023-07-21

**Authors:** Sourav Mondal, Kinkar Chandra Das

**Affiliations:** 1 Department of Mathematics, Sungkyunkwan University, Suwon 16419, Republic of Korea; sourav.94@skku.edu; 2 Department of Mathematics, SRM Institute of Science and Technology, Kattankulathur 603203, Tamil Nadu, India

**Keywords:** entropy, chemical graph theory, molecular graph, topological index, QSPR analysis

## Abstract

Graph entropy plays an essential role in interpreting the structural information and complexity measure of a network. Let *G* be a graph of order *n*. Suppose $d_{G}\left(v_{i}\right)$ is degree of the vertex $v_{i}$ for each i=1,2,…,n. Now, the *k*-th degree-based graph entropy for *G* is defined as $I_{d,k}\left(G\right)=-\sum_{i=1}^{n}\left(\frac{d_{G}{\left(v_{i}\right)}^{k}}{\sum_{j=1}^{n}d_{G}{\left(v_{j}\right)}^{k}}\;log\;\frac{d_{G}{\left(v_{i}\right)}^{k}}{\sum_{j=1}^{n}d_{G}{\left(v_{j}\right)}^{k}}\right),$ where *k* is real number. The first-degree-based entropy is generated for k=1, which has been well nurtured in last few years. As $\sum_{j=1}^{n}d_{G}{\left(v_{j}\right)}^{k}$ yields the well-known graph invariant first Zagreb index, the $I_{d,k}$ for k=2 is worthy of investigation. We call this graph entropy as the second-degree-based entropy. The present work aims to investigate the role of $I_{d,2}$ in structure property modeling of molecules.

## 1. Introduction

Graph theory has developed into a powerful mathematical tool in a wide range of disciplines, including operational research, chemistry, genetics, and linguistics, as well as electrical engineering, geography, sociology, and architecture. In addition, it has grown into a useful field of mathematics on its own. Using a diagram composed of a collection of points with lines connecting specific pairs of these points, many real-world situations can be simply explained. Chemists work with graphs on a daily basis because almost all chemistry interactions are carried out through the graphic representation of compounds and reactions. Chemical graph theory appears to be the natural language of chemistry through which chemists communicate. One of the important tools in this area is the graph invariant, which is any property of molecular graph that remains unchanged under graph isomorphism. Numerous kinds of graph invariants have appeared in the literature based on different graph parameters. One such important parameter is the degree of vertex, which is defined as the number of incident edges. For a molecular graph, it represents the valency of the corresponding atom. For degree-based invariants, readers are referred to the article [1] and the references cited therein. The present work deals with degree-based graph entropy. Shannon et al. [2] put forward the concept of entropy in 1949, and it is now one of the most significant measures in information theory as an indicator of the randomness of information content. This idea was imposed on graphs in 1955 [3], employing certain probability distributions associated with the automorphisms of graphs. Graph entropies vary depending on the probability distributions set on the graph. Dehmer’s graph entropies [4] based on information functionals are one of the highlights and have led to many significant research insights in the fields of information science, graph theory, and network science. Entropy corresponding to the independent sets and the matching of graphs is investigated in [5]. Bounds of such entropy measures are illustrated in 2020 [6]. The distance between two vertices is employed to design a new type of entropy in [7] whose upper bounds are set up by Ilić and Dehmer [8]. Cao et al. [9] investigated numerous attributes of entropy measure formulated on information functional by considering degree powers of graphs. To obtain insight about the quantity degree power, readers are referred to [1,10,11]. For further knowledge on graph entropy, see survey work [12].

For probability distribution $\alpha =(\alpha_{1},\alpha_{2},\ldots ,\alpha_{n})$, the entropy I(α) due to Shannon is defined as

$$I\left(\alpha \right)=-\sum_{i=1}^{n}\alpha_{i}\;log\;\alpha_{i}.$$

Let G be a finite, undirected and connected graph with vertex set $V={\left\{v_{i}\right\}}_{i=1}^{n}$. For each vertex $v_{i}$, Dehmer [4] defined

$$\alpha_{i}=\frac{\phi (v_{i})}{\sum_{j=1}^{n}\phi \left(v_{j}\right)},$$

so that the entropy of *G* based on ϕ is formulated as

(1) $$I_{\phi }\left(G\right)=-\sum_{i=1}^{n}\left(\frac{\phi (v_{i})}{\sum_{j=1}^{n}\phi \left(v_{j}\right)}\;log\;\frac{\phi (v_{i})}{\sum_{j=1}^{n}\phi \left(v_{j}\right)}\right).$$

Now, different entropy can be generated by varying ϕ. Cao et al. [9] set $\phi \left(v_{i}\right)=d_{G}{\left(v_{i}\right)}^{k}$, where $d_{G}\left(v_{i}\right)$ stands for the degree of vertex $v_{i}$, and proposed the *k*-th degree-based graph entropy as follows

(2) $$I_{d,k}\left(G\right)=-\sum_{i=1}^{n}\left(\frac{d_{G}{\left(v_{i}\right)}^{k}}{\sum_{j=1}^{n}d_{G}{\left(v_{j}\right)}^{k}}\;log\;\frac{d_{G}{\left(v_{i}\right)}^{k}}{\sum_{j=1}^{n}d_{G}{\left(v_{j}\right)}^{k}}\right),$$

where *k* is real number. For k=1, the entropy $I_{d,1}$ is named as the first degree-based entropy in [9] and extremal graphs for different classes are characterized. For more works on such measures, see [13,14,15]. For k=2, the quantity $\sum_{i=1}^{n}d_{G}{\left(v_{j}\right)}^{k}$ gives the first Zagreb index [1,10,16,17], which is well-known and mostly used in chemical graph theory. Thus, it is worthwhile to investigate the entropy measure $I_{d,2}$. We call it the second-degree-based entropy.

Predictive quantitative structure–property relationship (QSPR) models play an essential role in the design of purpose-specific fine chemicals such as pharmaceuticals. It is usually very costly to test a compound using a wet lab, but the QSPR study allows that cost to be reduced. Topological indices plays an important role in establishing structure property relationship of molecule. For some recent works on this analysis, readers are referred to [18,19,20,21]. The ultimate goal of the present work is to investigate the role of second-degree-based graph entropy $I_{d,2}$ in structure–property modeling of molecules.

## 2. Application Potential of Entropy

Topological indices abound and continue to grow in number. The majority of them are handled mathematically, lacking any sense of their chemical value. As a result, a collection of beneficial components was assembled to aid in picking of a pertinent molecular descriptor from a vast pool of candidates. Among the numerous qualities specified is the ability to anticipate the properties and activities of molecules. For the purpose of looking into the predicting ability of topological indices, quantitative structure–property relationship analysis is usually performed on theoretical attributes and experimental measures of some benchmark chemicals. The entropy-based indices are nurtured well in mathematical chemistry from a mathematical standpoint. Our aim is to illustrate the chemical connection of the entropy corresponding to the first Zagreb index. First, we consider the octane isomers as benchmark datasets. As octanes contain no cycles, we then take into account some hydrocarbons having cycles as a substructure.

**Figure 1:**
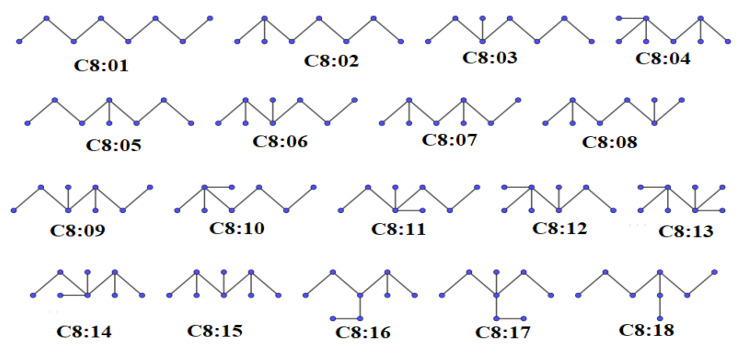
Molecular graph representations of octanes.

The molecular graph representations of octane isomers are displayed in Figure 1. The numerical values of different properties and the $I_{d,2}$ index are reported in Table 1.

The $I_{d,2}$ index is found to have a significant correlation with entropy (*S*), enthalpy of vaporization (HVAP), standard enthalpy of vaporization (DHVAP), and acentric factor (AF). We investigate the following relation to examine the potential of $I_{d,2}$.

(3) $$\begin{array}{c} P=C_{1}\;\left(\pm 2\times E_{1}\right)\;I+C_{2}\;\left(\pm 2\times E_{2}\right), \end{array}$$

where *P*, *I*, $C_{1}$, $C_{2}$, and $E_{i}^{\prime }s$ represent property, index, slope, intercept, and errors, respectively. Performed regression analysis also contains standard error (SE), the F-test (*F*), and the significance *F* (SF), in addition to *R*, to judge more accurately. For *S* and AF, $I_{d,2}$ yields the following structure–property relationships.

(4) $$\begin{array}{c} S=24.782\left(\pm 4.577\right)I_{d,2}+64.242\left(\pm 7.642\right),\\ R^{2}=0.879,\;\;\;SE=1.613,\;\;\;F=117.276,\;\;\;SF=8.97\times 10^{-9} \end{array}$$

(5) $$\begin{array}{c} HVAP=10.637\left(\pm 2.61\right)I_{d,2}+51.491\left(\pm 4.358\right),\\ R^{2}=0.806,\;\;\;SE=0.92,\;\;\;F=66.415,\;\;\;SF=4.36\times 10^{-7} \end{array}$$

**Figure 2:**
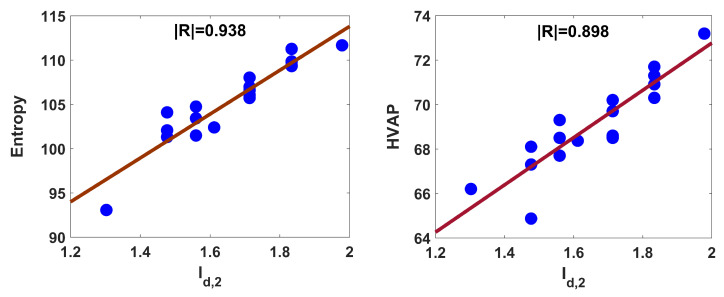
Linear fitting of $I_{d,2}$ with entropy and HVAP for octanes.

The linear fittings of relations (4) and (5) are shown in Figure 2.

For DHVAP and AF, the regression relation (3) takes following form.

(6) $$\begin{array}{c} DHVAP=2.112\left(\pm 0.376\right)I_{d,2}+5.618\left(\pm 0.627\right),\\ R^{2}=0.888,\;\;\;SE=0.132,\;\;\;F=126.325,\;\;\;SF=5.28\times 10^{-9} \end{array}$$

(7) $$\begin{array}{c} AF=0.2\left(\pm 0.026\right)I_{d,2}+0.003\left(\pm 0.044\right),\\ R^{2}=0.935,\;\;\;SE=0.009,\;\;\;F=229.253,\;\;\;SF=6.64\times 10^{-11}. \end{array}$$

**Figure 3:**
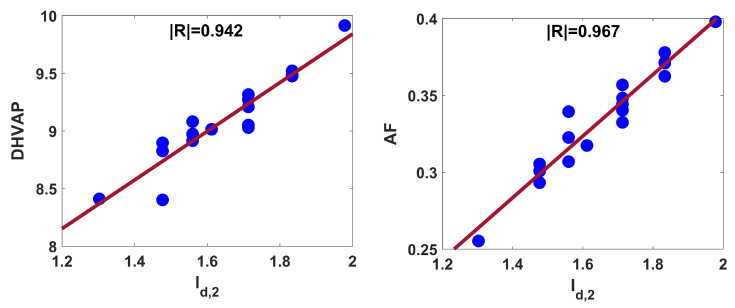
Linear fitting of $I_{d,2}$ with DHVAP and AF for octanes.

The strength of structure property relationships (6) and (7), is displayed in Figure 3. The blue circles in Figure 2 and Figure 3 are the points (x, y), where x and y represent the $I_{d,2}$ and property for octanes, respectively, and the red line indicates the regression line.

From the $R^{2}$ values, one can say that the data variances for *S*, HVAP, DHVAP, and AF are 88%, 81%, 89%, and 94%, respectively. The blue circles for AF in Figure 3 are closure to the regression line compared to other frames. As the SE value decreases, the regression relation becomes strong. Each of the aforesaid equations yields small SE, AF especially is significantly low. The model’s consistency boosts as the F-value rises. The F-value in model (7) is comparatively high. The model is regarded as statistically reliable when the SF value is less than 0.05. In each case, the SF value is significantly less than 0.05. Thus, one can conclude that the second-degree-based entropy exerts better performance in explaining acentric factor compared to *S*, HVAP, and DHVAP. Now, we will perform external validation for the constructed model in case of AF. The nonane isomer is considered here as an external data set. The set is divided into train and test sets in the ratio 80:20 by means of python scikit learn machine learning module. The train set is considered to generate the model, which is validated by the test set.

(8) $$\begin{array}{c} AF=0.252\left(\pm 0.039\right)I_{d,2}-0.087\left(\pm 0.071\right),\\ R^{2}=0.86,\;\;\;SE=0.016,\;\;\;F=159.886,\;\;\;SF=1.3\times 10^{-12}. \end{array}$$

**Figure 4:**
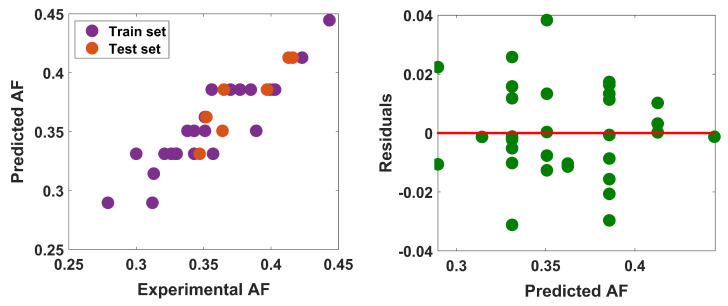
Experimental vs. predicted AF and residual plot.

The relation (8) expresses the structure–property relationship in the train set, where the data variance is 86%. Plotting of predicted data against experimental data and random scattering in residual plot (see Figure 4) ensure that the model on training set is well aligned and consistent. The data variance on test set is 82%, which confirms that the external validation is meaningful.

Now to compare the performance of $I_{d,2}$ with some well known degree-based indices, we correlate first ($M_{1}$) and second ($M_{2}$) Zagreb indices, forgotten index (*F*), inverse sum indeg index (ISI), symmetric division degree index (SDD), sum connectivity index (SCI), and inverse Randić index (RR). The absolute correlation coefficients of aforesaid indices with *S*, HVAP, DHVAP, and AF for octanes are reported in Table 2. In case of *S*, the $I_{d,2}$ performs better than ISI, SDD, SCI, and RR. The present invariant outperforms $M_{1}$, *F*, $M_{2}$, ISI, and RR for HVAP. The correlation of $I_{d,2}$ with DHVAP is better than that of $M_{1}$, *F*, $M_{2}$, ISI, and RR. In case of AF, the current descriptor outperforms *F*, ISI, SDD, and SCI.

**Figure 5:**
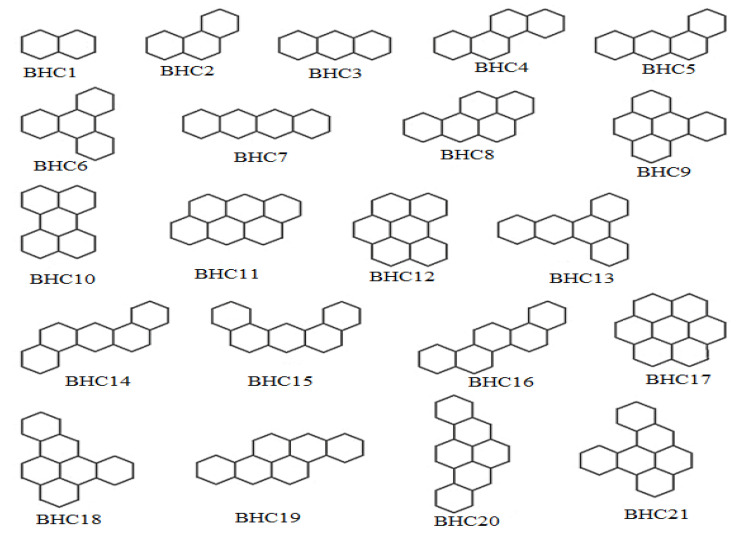
Molecular graphs of benzenoid hydrocarbons.

Now, we consider some benzenoid hydrocarbons (BHCs) for investigation. The molecular structures of BHCs are shown in Figure 5.

The second-degree-based entropy is observed to correlate well with the boiling point (BP) of benzenoid hydrocarbons. The BP and $I_{d,2}$ values are reported in Table 3.

The $I_{d,2}$ index is also noticed to have significant correlation with the π-electron energy of benzenoid hydrocarbons. The regression relations for BP and $E_{\pi }$ is as follows:

(9) $$\begin{array}{c} BP=447.594\left(\pm 28.633\right)I_{d,2}-808.089\left(\pm 6.474\right),\\ R^{2}=0.98,\;\;\;SE=14.157,\;\;\;F=977.456,\;\;\;SF=8.38\times 10^{-18} \end{array}$$

(10) $$\begin{array}{c} E_{\pi }=24.942\left(\pm 1.6\right)I_{d,2}-44.039\left(\pm 4.65\right),\\ R^{2}=0.981,\;\;\;SE=0.792,\;\;\;F=970.691,\;\;\;SF=8.94\times 10^{-18}. \end{array}$$

**Figure 6:**
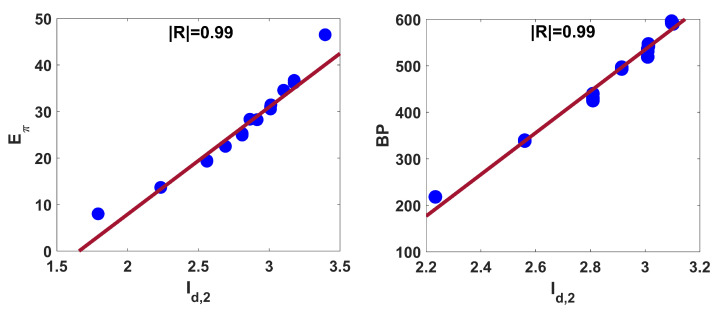
Linear fitting of $I_{d,2}$ with $E_{\pi }$ and BP for benzenoid hydrocarbons.

From relations (9) and (10), we can say that 94% and 98% of observations fit the models related to BP and $E_{\pi }$, respectively. The corresponding linear fittings are shown in Figure 6. For comparative purposes, we correlate some well-known degree-based indices with boiling point and π-electron energy for benzenoid hydrocarbons. The correlation coefficients displayed in Table 4 yield that $I_{d,2}$ outperforms some of those well-established indices.

**Figure 7:**
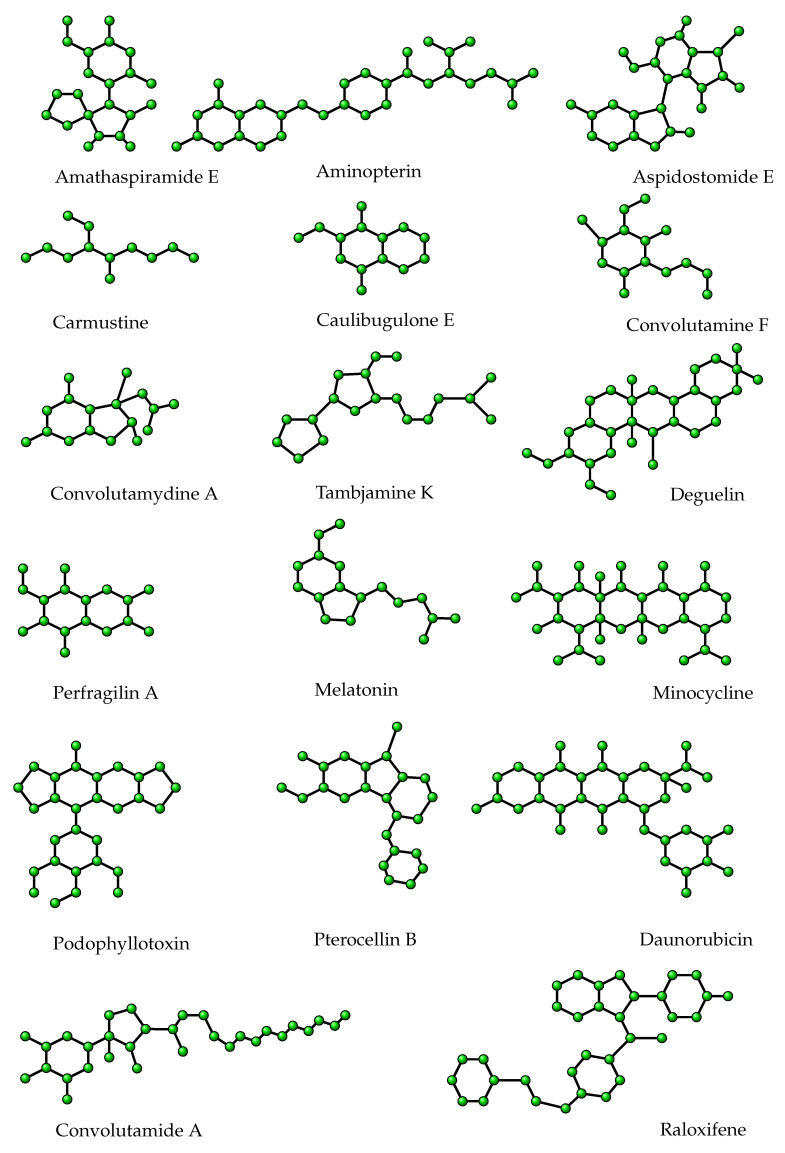
Molecular graphs of some chemicals useful in drug preparation.

Now, we consider some molecular graphs having cyclic substructure which are useful in drug preparation. These compounds include Aminopterin, Aspidostomide E, Carmustine, Caulibugulone E, Convolutamine F, Convolutamydine A, Tambjamine K, Deguelin, Perfragilin A, Melatonin, Minocycline, Podophyllotoxin, Pterocellin B, Daunorubicin, Convolutamide A, Raloxifene. The molecular graphs of these structures are displayed in Figure 7. The experimental and theoretical measures for these compounds are reported in Table 5.

The $I_{d,2}$ index is noticed to perform well for molar refraction (MR) and boiling point (BP) for the aforesaid structures. Corresponding regression relations are as follows:

(11) $$\begin{array}{c} BP=381.182\left(\pm 123.068\right)I_{d,2}-538.567\left(\pm 360.377\right),\\ R^{2}=0.733,\;\;\;SE=89.762,\;\;\;F=38.374,\;\;\;SF=2.34\times 10^{-5} \end{array}$$

(12) $$\begin{array}{c} MR=74.224\left(\pm 13.249\right)I_{d,2}-122.652\left(\pm 38.797\right),\\ R^{2}=0.899,\;\;\;SE=9.66,\;\;\;F=125.5426,\;\;\;SF=2.24\times 10^{-8}. \end{array}$$

**Figure 8:**
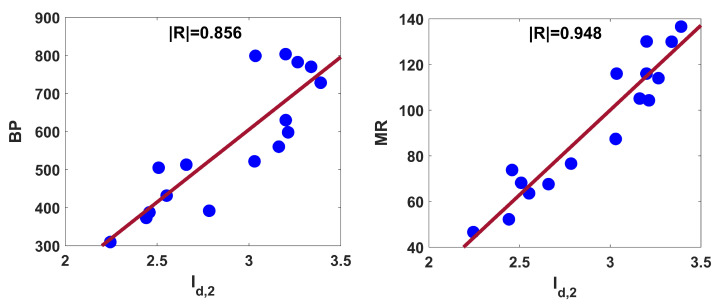
Linear fitting of $I_{d,2}$ with BP and MR for some structures displayed in Figure 7.

Equations (11) and (12) reveal that the coefficient of determination for BP and MR are 73% and 90%, respectively. The linear fittings of the aforementioned structure–property relationship are shown in Figure 8.

To compare the present descriptor with $M_{1}$, *F*, $M_{2}$, ISI, SDD, SCI, and RR, we correlate the degree-based indices with BP and MR for chemicals displayed in Figure 7. The correlation coefficients reported in Table 6 imply that $I_{d,2}$ performs better than some of the well-known and most-used indices.

Now, to check the relationship of $I_{d,2}$ with well-known degree-based indices, we correlate $I_{d,2}$ with SCI, ISI, RR, SDD, $M_{1}$, $M_{2}$ and *F*. The absolute correlation coefficients of $I_{d,2}$ with aforesaid indices are reported in Table 7 for decane isomers. It shows that first Zagreb index and forgotten topological index are strongly correlated with $I_{d,2}$. Thus, there is a possibility of having a strong mathematical relation between them.

## 3. Concluding Remarks

The impact of the entropy on structure property modeling corresponding to the first Zagreb index has been investigated in this work. The $I_{d,2}$ index has been found to have a significant predictive potential for physiochemical properties of octane isomers. The linear relation of $I_{d,2}$ with entropy, enthalpy of vaporization, standard enthalpy of vaporization, and acentric factor has been found to be satisfactory. Especially, the performance of $I_{d,2}$ in explaining AF is remarkable. An external validation using nonane isomers confirms this claim. The present entropy has been observed to model boiling point and π-electron energy of benzenoid hydrocarbons with powerful accuracy. The $I_{d,2}$ is also capable of explaining boiling point and molar refraction of some compounds useful in drug generation. The second-degree-based entropy performs better than some well-known and commonly used degree-based indices for three data sets. This empirical study is expected to be performed on other data sets in the future, including aromatic and hetero-aromatic amines, polychlorobiphenyls, poly-arometic hydrocarbons, and so on. The strong correlation of $I_{d,2}$ with $M_{1}$ and *F* indicates that there may be a strong mathematical connection between them, which could be considered as a future research direction.

## Figures and Tables

**Table 1:** Different properties and $I_{d,2}$ index for octane isomers.

Octanes	*S*	HVAP	DHVAP	AF	$I_{d,2}$
C8:01	111.67	73.19	9.915	0.3979	1.9784
C8:02	109.84	70.3	9.484	0.3779	1.8338
C8:03	111.26	71.3	9.521	0.371	1.8337
C8:04	109.32	70.91	9.483	0.3715	1.8338
C8:05	109.43	71.7	9.476	0.3625	1.8338
C8:06	103.42	67.7	8.915	0.3394	1.5596
C8:07	108.02	70.2	9.272	0.3482	1.7132
C8:08	106.98	68.5	9.029	0.3442	1.7132
C8:09	105.72	68.6	9.051	0.3568	1.7132
C8:10	104.74	68.5	8.973	0.3225	1.5596
C8:11	106.59	70.2	9.316	0.3403	1.7132
C8:12	106.06	69.7	9.209	0.3324	1.7132
C8:13	101.48	69.3	9.081	0.3067	1.5596
C8:14	101.31	67.3	8.826	0.3008	1.4769
C8:15	104.09	64.87	8.402	0.3054	1.4769
C8:16	102.06	68.1	8.897	0.2932	1.4769
C8:17	102.39	68.37	9.014	0.3174	1.6118
C8:18	93.06	66.2	8.41	0.2552	1.3028

**Table 2:** The absolute correlation coefficients of some degree-based indices with *S*, HVAP, DHVAP, and AF for octane isomers.

	$M_{1}$	*F*	$M_{2}$	ISI	SDD	SCI	RR
*S*	0.954	0.953	0.942	0.636	0.909	0.923	0.953
HVAP	0.886	0.872	0.728	0.271	0.928	0.932	0.812
DHVAP	0.936	0.924	0.812	0.384	0.953	0.961	0.881
AF	0.973	0.965	0.986	0.733	0.901	0.929	0.995

**Table 3:** Boiling point, π-electron energy, and invariants for BHC.

Compounds	BP	$E_{\pi }$	$I_{d,2}$	Compounds	BP	$E_{\pi }$	$I_{d,2}$
BHC1	2.2338	218	13.6832	BHC12	3.0121	542	31.4251
BHC2	2.5603	338	19.4483	BHC13	3.0096	535	30.9418
BHC3	2.5603	340	19.3137	BHC14	3.0096	536	30.8805
BHC4	2.8094	431	25.1922	BHC15	3.0096	531	30.8795
BHC5	2.8094	425	25.1012	BHC16	3.0096	519	30.9432
BHC6	2.8094	429	25.2745	BHC17	3.1021	590	34.5718
BHC7	2.8094	440	24.9308	BHC18	3.0974	592	34.0646
BHC8	2.9146	496	28.222	BHC19	3.097	596	33.1892
BHC9	2.9146	493	28.3361	BHC20	3.0974	594	33.9542
BHC10	2.9146	497	28.2453	BHC21	3.0974	595	34.0307
BHC11	3.0121	547	31.2529				

**Table 4:** The absolute correlation coefficients of some degree-based indices with BP and $E_{\pi }$ for BHC.

	$M_{1}$	*F*	$M_{2}$	ISI	SDD	SCI	RR
BP	0.988	0.979	0.975	0.987	0.996	0.997	0.988
$E_{\pi }$	0.993	0.985	0.982	0.992	0.999	0.999	0.993

**Table 5:** Boiling point, molar refraction and graph invariant for structures displayed in Figure 7.

Compounds	BP	MR	$I_{d,2}$	Compounds	BP	MR	$I_{d,2}$
Aminopterin	782.27	114	3.2656	Perfragilin A	560.1	105.1	3.1628
Aspidostomide E	798.8	116	3.0354	Melatonin	512.8	67.6	2.6596
Carmustine	309.6	46.6	2.2456	Minocycline	803.3	116	3.1999
Caulibugulone E	373	52.2	2.4413	Podophyllotoxin	431.5	63.6	2.5523
Convolutamine F	629.9	130.1	3.2006	Pterocellin B	597.9	104.3	3.2134
Convolutamydine A	387.7	73.8	2.4587	Daunorubicin	521.6	87.4	3.0304
Tambjamine K	504.9	68.2	2.5088	Convolutamide A	728.2	136.6	3.3907
Deguelin	770	130	3.3387	Raloxifene	391.7	76.6	2.784

**Table 6:** The absolute correlation coefficients of some degree-based indices with BP and MR for some structures depicted in Figure 7.

	$M_{1}$	*F*	$M_{2}$	ISI	SDD	SCI	RR
BP	0.869	0.835	0.834	0.862	0.885	0.874	0.866
MR	0.888	0.818	0.83	0.894	0.899	0.947	0.891

**Table 7:** The absolute correlation coefficients of $I_{d,2}$ with some degree-based indices for decane isomers.

	SCI	ISI	RR	SDD	$M_{1}$	$M_{2}$	*F*
$I_{d,2}$	0.576	0.939	0.96	0.944	0.992	0.683	0.994

## Data Availability

Not applicable.
